# The influence of menopause on multiple sclerosis

**DOI:** 10.1111/ene.16566

**Published:** 2024-11-27

**Authors:** Cecilia Smith Simonsen, Heidi Øyen Flemmen, Line Broch, Harald Myklebust, Pål Berg‐Hansen, Cathrine Brunborg, Elisabeth Gulowsen Celius

**Affiliations:** ^1^ Department of Neurology Vestre Viken Hospital Trust Drammen Norway; ^2^ Department of Neurology Hospital Telemark HF Skien Norway; ^3^ Department of Neurology Oslo University Hospital Oslo Norway; ^4^ Institute of Clinical Medicine University of Oslo Oslo Norway; ^5^ Oslo Centre for Biostatistics and Epidemiology, Research Support Services Oslo University Hospital Oslo Norway

**Keywords:** multiple sclerosis, menopause, late onset MS (LOMS), EDSS progression, annual relapse rate

## Abstract

**Introduction:**

One third of the multiple sclerosis (MS) population consists of peri‐ or postmenopausal women. Many symptoms of menopause overlap those of MS. Some studies show increased speed of disability progression after menopause, while others indicate an unaltered trajectory.

**Objective:**

Determine the association between menopause and MS disease course.

**Methods:**

Cohort study with clinical data collected prospectively. Self‐reported age of menopause, smoking and parity collected retrospectively.

**Results:**

We included 559 peri−/postmenopausal women and 386 similarly aged men. There was no significant difference in EDSS progression (slope coef 0.03, 0.02–0.08, *p* = 0.208) or annual relapse rate (ARR) (0.10, 0.29–0.10, *p* = 0.324) in the 5 years before and after menopause. Women's EDSS progressed slower than men's after menopause (coef −0.02, 95% CI −0.04 to −0.002, *p* = 0.032), but there was no difference in ARR [coef. −0.01, 95% CI −0.04 to −0.01, *p* = 0.262]. Women who reached menopause before *MS onset* had shorter time to diagnosis than women who reached menopause after onset (3.1 years (3.1) vs. 7.4 years (8.1), *p* < 0.001). Women who reached menopause before *diagnosis* had an even longer time to diagnosis (8.8 (9.3) vs. 5.5 (6.3), *p* < 0.001).

**Conclusions:**

There were no significant differences in EDSS progression or ARR before and after menopause. In fact, men progressed faster than women, suggesting there is no negative impact of menopause on disease progression, as measured by EDSS and relapses.

## INTRODUCTION

Multiple sclerosis (MS) is an inflammatory, demyelinating and neurodegenerative disease of the central nervous system. MS is most common in women, with a 3:1 ratio in the reproductive years and an observed increase in female incidence over the past couple of decades [1]. However, before puberty [2] and after menopause [3], this gender disparity evens out. The relapse rate in women declines in pregnancy, there is increased disease activity the first 3 months postpartum [4] and women have more inflammatory disease activity in terms of relapses than men up to the age of menopause [5]. Men are more likely to have progressive disease at onset, report a greater proportion of motor relapses and have higher rates of cognitive impairment and atrophy, compared to women [6]. Finally, women are over‐represented in most autoimmune diseases [7]. All these implicates an influence of sex hormones on both MS susceptibility and disease course [8]. High levels of oestrogen are primarily anti‐inflammatory, inhibiting production and signalling of pro‐inflammatory cytokines, as well as inhibiting natural killer cell activation. Conversely, low levels of oestrogen most likely promote a pro‐inflammatory environment [9]. In addition, female sex hormones may have a neuroprotective effect on the brain, with earlier age at menopause being associated with faster decline in cognition [10, 11]. Ovarian ageing, as measured by anti‐Müllerian hormone (AMH), a hormone that is strongly correlated with antral follicle counts and is predictive of age at menopause, is associated with greater disability and grey matter loss in women with MS, independent of chronological age and disease duration [12].

Menopause, the permanent cessation of menstrual cycles due to the loss of ovarian follicular activity and a drop in levels of oestrogen, typically occurs between the ages of 45 years and 56 years. However, menopause is a transition that takes place over time, and therefore can begin well before the loss of menstruation [13]. An estimated 30% of the current MS population consists of peri‐ or postmenopausal women and many symptoms of menopause overlap those of MS [14]. Given lower lifetime exposure to oestrogen is associated with dementia [15], the drop in oestrogen may increase the neurodegeneration seen in pwMS. Some studies show increased speed of disability progression after menopause [16, 17], while others indicate a similar trajectory [18, 19]. Relapses diminish with age, but there may be a significant decrease in annual relapse rate (ARR) at the time of menopause [16, 18], though the data are not consistent [20]. Other autoimmune diseases often have an increase in inflammatory activity around menopause [7]. Considering the anti‐inflammatory effects of high levels of oestrogen and the pro‐inflammatory effects of low oestrogen, the impact of the oestrogen decline observed during menopause on disease relapses remains uncertain. There is a need for more research to disentangle the effects of menopause on symptoms and the disease course in people with MS (pwMS) [21]. In a recent review focusing on two large surveys, menopause was identified as a significant priority area among all types of stakeholders, with a particular emphasis on how perimenopause and menopause influence disease activity [22].

## METHODS

### Population

This is a cohort study from the BOT‐MS, a registry containing detailed information on a well‐defined MS population in the three counties Buskerud, Oslo and Telemark in Norway. These hospitals serve a population of 1.17 million people. We included all female pwMS who replied that they had reached menopause. These pwMS ranged from 41 to 81 years of age. In addition, we included all men in the cohort in the same age group (41–81 years at inclusion) as a control group. We included both pwMS with relapsing and progressive disease at onset.

### Outcomes

**FIGURE 1:**
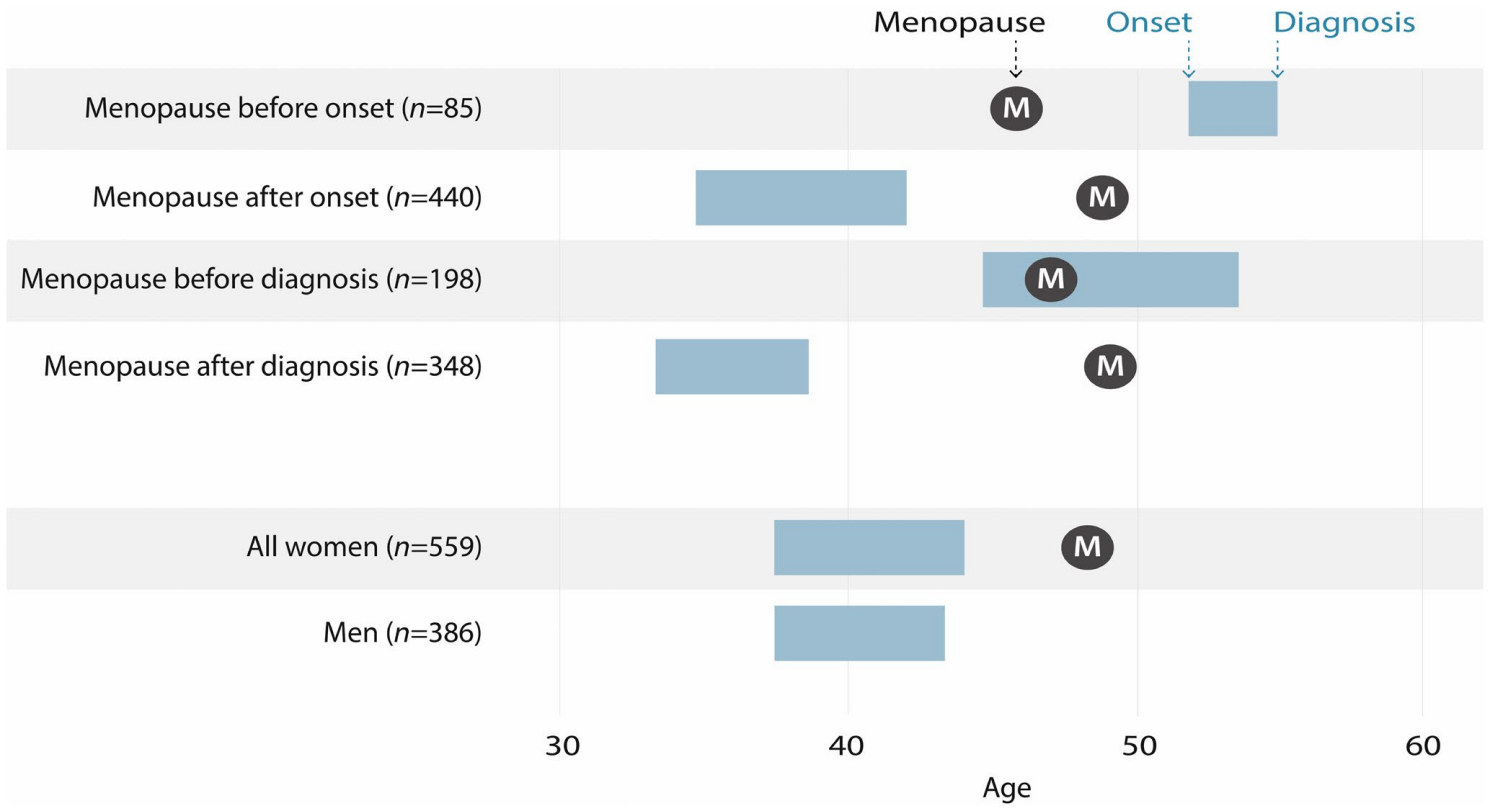
Mean age at onset, diagnosis and menopause in those with menopause before or after diagnosis, those with menopause before or after onset and in the complete population.

**FIGURE 2:**
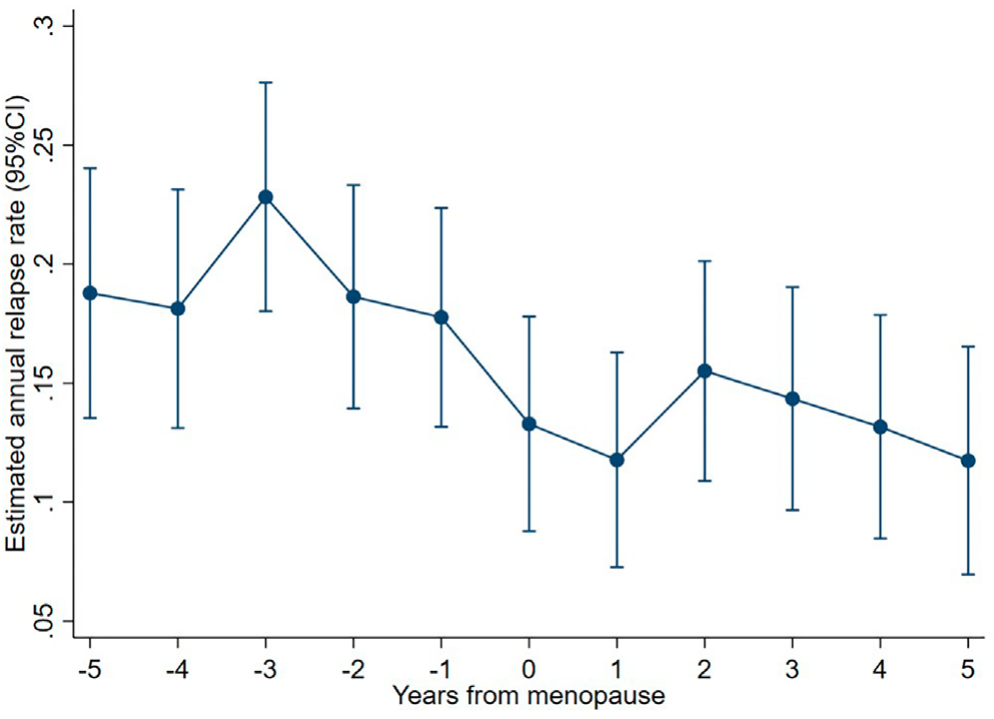
Unadjusted annual relapse rate 5 years before and after menopause (year 0).

Data were recorded prospectively until 31 December 2017, but retrieved retrospectively by three neurologists specialised in MS, between January and December 2018. Detailed information on the database and data collection has previously been published [23]. We collected information on disease onset and diagnosis, treatments, relapses and expanded disability status scale (EDSS) [24] at as many time points as possible. EDSS progression and relapses are presented in all women with MS in the cohort, irrespective of timing of menopause. Chronological age was defined as age at the time of inclusion (in 2018) and disease duration was defined as time from symptom onset to 2018. The multiple sclerosis severity score (MSSS) adds the element of disease duration to the EDSS and is designed to provide a measure of disease severity [25]. We calculated the MSSS using the duration of MS from time of onset and the EDSS score nearest to prevalence date. We have only used MSSS as additional information in Figures 1 and 2, MSSS was not used in the regression analyses. The pwMS provided information on year of menarche and menopause, parity and smoking habits through a validated questionnaire (ECTRIMS Online Library. Flemmen H. 09/12/19; 279,125; P765). We did not differentiate between natural or surgical menopause, as we did not have access to this information. Disease onset was defined as the first definite MS symptom onset. Late‐onset MS (LOMS) was defined as onset of MS symptoms at age 50 years or older. Moderate efficacy disease modifying therapies (DMTs) were defined as interferons, glatiramer acetate, teriflunomide and dimethyl‐fumarate, and high efficacy DMTs were defined as natalizumab, fingolimod, alemtuzumab, rituximab and stem cell therapy. DMTs were available and reimbursed since market access in Europe.

### Statistics

We used IBM SPSS Statistics 29.0 (IBM Corp., Armonk, NY, USA) and Stata 17 (Stata Corp. LLC, College Station, TX, USA) for data analysis. Between groups, differences in continuous variables were tested with Student t‐test for normally distributed data and Mann–Whitney U‐test for very skewed data. The chi‐square test for contingency tables was used to detect associations between categorical variables. Linear regression using year of birth as a continuous, independent variable was used to detect a trend in age of menopause. Linear mixed effects models were fitted to investigate the association between the EDSS 5 years before and after menopause to account for repeated measures by person with MS and to adjust for covariates. We modelled time by using a piecewise linear spline with a knot at time of menopause. Time and possible covariates were treated as fixed effects. All models included random intercept and slope. The following covariates were included: chronological age, disease duration, smoking, ever used high efficacy therapy and parity. We also tried adding treatment with any DMT, months on high efficacy therapy and months on any DMT, but these did not change the results and was not included in the final models. Multicollinearity was checked for using variable inflation factor (VIF). All the covariates had a VIF just over 1. Logistic mixed effects model were fitted to investigate the proportion of pwMS with relapses 5 years before and after menopause using the same procedures as described above. Linear mixed effect models was used to investigate if longitudinal changes in EDSS progression differed between men and women to account for repeated measures by patients. Time, gender and time‐by‐gender interaction were included as fixed effect in the models. Age at EDSS was used as the time parameter in the models, with 41 years as a starting point. Similarly, logistic mixed effects models was used to investigate longitudinal changes in proportion of patients with relapses differed between men and women. All *p*‐values were two‐sided and a 5% significance level was used.

### Ethical considerations

All participants provided written, informed consent. The Regional Ethics Committee (REK 2015/670) and the local Data Protection Officer approved the study under the condition that strict privacy concerns were respected. Consequently, data will not be made publicly available and specific requests regarding data sharing should be directed to the corresponding author.

## RESULTS

### PwMS demographics

The BOT‐MS registry comprises 3951 people with MS. The 2512 living pwMS were invited to participate in the study and 1573 completed the questionnaire (63% response rate). Of these, 1102 were women and 559 reported that they had reached menopause, constituting 51% of female responders. We included 386 men as a control group. Table 1 shows the pwMS demographics. Mean age of self‐reported menopause was 48.3 years (range: 35–60 years, median: 49.0 years). The male patients were more likely to have pyramidal symptoms and progressive disease at onset and were more likely to have been treated with high efficacy therapy.

**TABLE 1:** Demographics and clinical data.

	Menopausal women	Male control group	*p*
N	559	386	
Age, years mean (SD)	60.6 (7.9)	57.9 (9.7)	<0.001
EDSS at diagnosis, mean (SD)	2.7 (1.3)	2.6 (1.3)	0.181
Age at onset (SD)	37.4 (10.6)	37.4 (10.6)	0.464
Age at diagnosis (SD)	44.0 (10.2)	43.3 (10.1)	0.146
LOMS, %	14	13	0.582
Time from onset to diagnosis, years mean (SD)	6.7 (7.7)	5.8 (7.2)	0.047
Sensory symptoms at onset, %	35	30	0.127
Optic neuritis at onset, %	21	17	0.185
Pyramidal symptoms at onset, %	20	26	0.044
Progressive disease at onset, %	11	17	0.004
Multiple symptoms at onset, %	31	30	0.954
Present or past smokers, %	72	73	0.685
Live births, mean (range)	1.8 (0–6)	1.7 (0–6)	0.234
Ever treated, %	46	51	0.106
Ever treated with high efficacy DMT, %	18	24	0.037
Ever treated with mitoxantrone, %	3	2	0.243
Treatment at time of menopause, %	21	‐	
Menopause before diagnosis, %	36	‐	
Age at menopause, years mean (SD)	48.3 (4.6)	‐	
Age at menarche, years mean(SD)	13.1 (1.4)	‐	
Years from menarche to menopause mean (SD)	35.2 (4.7)	‐	
EDSS at menopause, median (IQR)	2.5 (2.5,4.5)	‐	
EDSS 2018 mean (SD)	4.0 (2.3)	4.2 (2.4)	0.221
MSSS 2018 mean(SD)	3.7 (2.7)	4.0 (2.7)	0.057

*Note*: EDSS expanded disability status scale, SD standard deviation, LOMS late onset MS (≥50 years), DMT disease modifying therapy and MSSS multiple sclerosis severity score.

Thirty percent of women started, stopped or changed DMTs the year they reached menopause. Table S1 details the use of disease modifying therapies in men versus women and in the peri−/postmenopausal period.

### Age of menopause

PwMS who had ever been treated with disease modifying therapy and pwMS who had given birth reached menopause significantly later. There was no difference in the mean age of menopause in the ever‐smoking group versus the never smoking groups or in the pwMS who had been treated with mitoxantrone versus not treated with mitoxantrone (Table S2).

### Menopause before or after disease onset and diagnosis

Table 2 and Figure 1 show the population divided into menopause before or after MS onset and MS diagnosis.

**TABLE 2:** Demographics and clinical findings in PwMS stratified by onset versus menopause on the left and by diagnosis versus menopause on the right.

	Menopause after MS onset	Menopause before MS onset	*p*	Menopause after MS diagnosis	Menopause before MS diagnosis	*p*
*N* (%)	440 (84)	85 (16)		348 (64)	198 (35)	
Age, years mean(SD)	58.8 (7.7)	63.3 (7.1)	<0.001	57.7 (7.4)	63.1 (7.3)	<0.001
EDSS at diagnosis, mean(SD)	2.6 (1.2)	2.9 (1.3)	0.095	2.5 (1.1)	2.9 (1.8)	0.003
Age at onset, mean (SD)	34.6 (9.0)	51.8 (5.8)	<0.001	33.2 (8.7)	44.7 (9.7)	<0.001
Age at diagnosis, mean (SD)	42.0 (9.4)	54.9 (6.2)	<0.001	38.6 (7.8)	53.6 (6.2)	<0.001
LOMS, %	3	69	<0.001	2	35	<0.001
Time from onset to diagnosis, mean (SD)	7.4 (8.1)	3.1 (3.1)	<0.001	5.5 (6.3)	8.8 (9.3)	<0.001
Sensory symptoms at onset, %	36	35	0.864	37	33	0.390
Optic neuritis at onset, %	22	12	0.035	21	19	0.609
Pyramidal symptoms at onset, %	18	29	0.032	17	24	0.044
Progressive disease at onset, %	9	18	0.011	8	17	0.002
Multiple symptoms at onset, %	30	34	0.469	32	29	0.625
Present or past smokers, %	73	68	0.370	74	67	0.071
Live births, mean (range)	1.7 (0–6)	1.7 (0–4)	0.372	1.7 (0–6)	1.7 (0–6)	0.991
Ever‐treated DMT, %	48	43	0.454	51	38	0.003
Ever‐treated high efficacy DMT, %	19	20	0.846	22	14	0.023
Treatment at time of menopause, %	23	0	‐	29	0	‐
Ever treated with mitoxantrone, %	3.2	2.4	0.684	3.5	2.5	0.547
Age at menopause, mean (SD)	48.8 (4.4)	45.8 (4.4)	<0.001	49.1 (4.5)	47.0 (4.4)	<0.001
Age at menarche, mean (SD)	13.1 (1.4)	13.0 (1.4)	0.150	13.1 (1.4)	13.0 (1.5)	0.318
Years from menarche to menopause, mean (SD)	35.6 (4.6)	32.8 (3.5)	<0.001	35.9 (4.7)	34.0 (4.5)	<0.001
Years from onset to menopause, mean (SD)	14.1 (9.0)	−6.0 (SD 4.9)	<0.001	15.9 (8.7)	2.3 (9.9)	<0.001
EDSS at menopause, mean (SD)	3.3 (2.0)	‐		3.4 (2.0)	2.7 (1.8)	0.276
EDSS 2018, mean (SD)	4.0 (2.3)	3.8 (2.0)	0.346	4.1 (2.4)	3.9 (2.1)	0.323
MSSS 2018, mean (SD)	3.5 (2.7)	4.5 (2.5)	<0.001	3.5 (2.8)	4.0 (2.5)	0.031

*Note*: EDSS expanded disability status scale, SD standard deviation, LOMS‐late onset MS (≥50 years), DMT disease modifying therapy and MSSS multiple sclerosis severity score.

In pwMS who reached *menopause before symptom onset*, time from onset to diagnosis was significantly shorter than for pwMS who reached menopause after symptom onset (3.1 years (SD 3.1) vs. 7.4 years (SD 8.1), *p* < 0.001). In addition, PwMS who reached menopause before symptom onset were more likely to have pyramidal symptoms and progressive disease at onset.

Conversely, pwMS who *reached menopause before being diagnosed with MS* had longer time from onset to diagnosis (8.8 years (SD 9.3) vs. 5.5 (SD 6.3), *p* < 0.001) compared to pwMS who reached menopause after diagnosis (see Table 3). They were also more likely to have a progressive disease at onset, had a higher EDSS at the time of diagnosis and a higher MSSS at the time of inclusion.

**TABLE 3:** Yearly EDSS progression and relapses, including slope difference, using mixed models analyses, unadjusted and adjusted^a^.

	Unadjusted			Adjusted^a^		
	Coef	95% CI	*p*	Coef	95% CI	*p*
Yearly relapses						
5 years before menopause slope	−0.08	−0.18 to 0.04	0.094	−0.07	−0.16 to –0.02	0.125
At menopause and 5 years after slope	−0.19	−0.32 to −0.05	0.007	−0.16	−0.29 to −0.03	0.017
Slope difference	−0.10	−0.30 to 0.09	0.295	−0.10	−0.28 to 0.10	0.363
Yearly EDSS progression						
5 years before menopause slope	0.09	0.06 to 0.12	<0.001	0.10	0.06 to 0.12	<0.001
At menopause and 5 years after slope	0.12	0.09 to 0.15	<0.001	0.11	0.08 to 0.14	<0.001
Slope difference	0.03	0.02 to 0.08	0.208	0.02	−0.02 to 0.07	0.315

^a^ Adjusted for disease duration, chronological age, smoking, high efficacy therapy and parity.

### Relapses and progression before and after menopause

**FIGURE 3:**
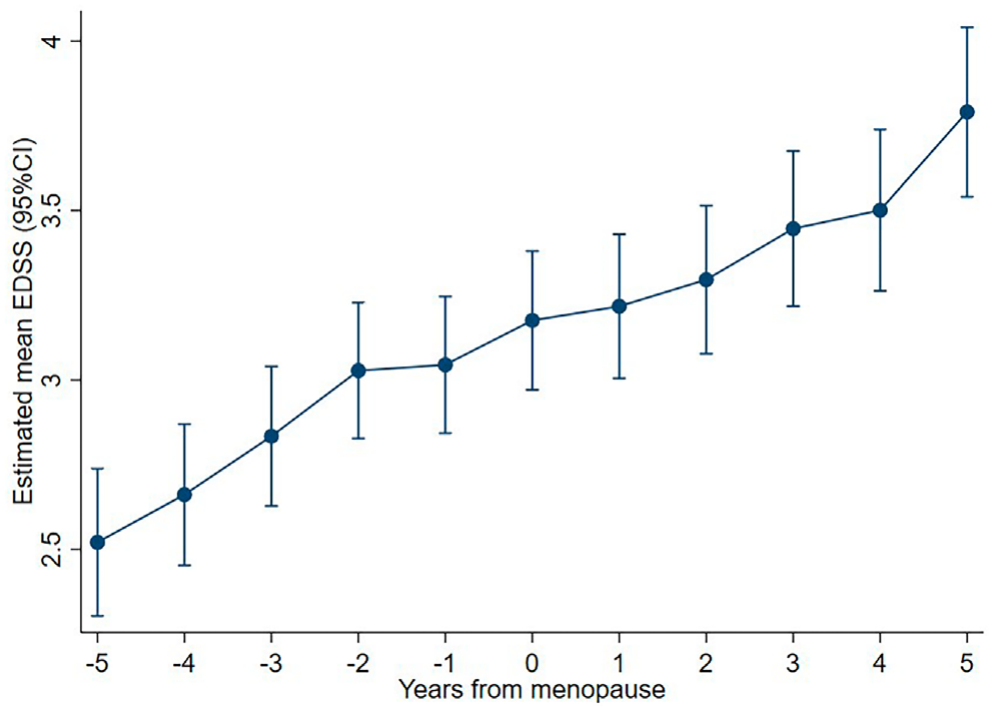
Unadjusted EDSS progression 5 years before and after menopause (year 0).

ARR decreased slightly more after menopause than before, but the difference was not significant (Table 3 and Figure 2). Similarly, yearly EDSS progression in the 5 years before menopause was lower than at perimenopause and the 5 years after, but this difference was not significant (Table 3 and Figure 3).

### Relapses and progression in men and women

**FIGURE 4:**
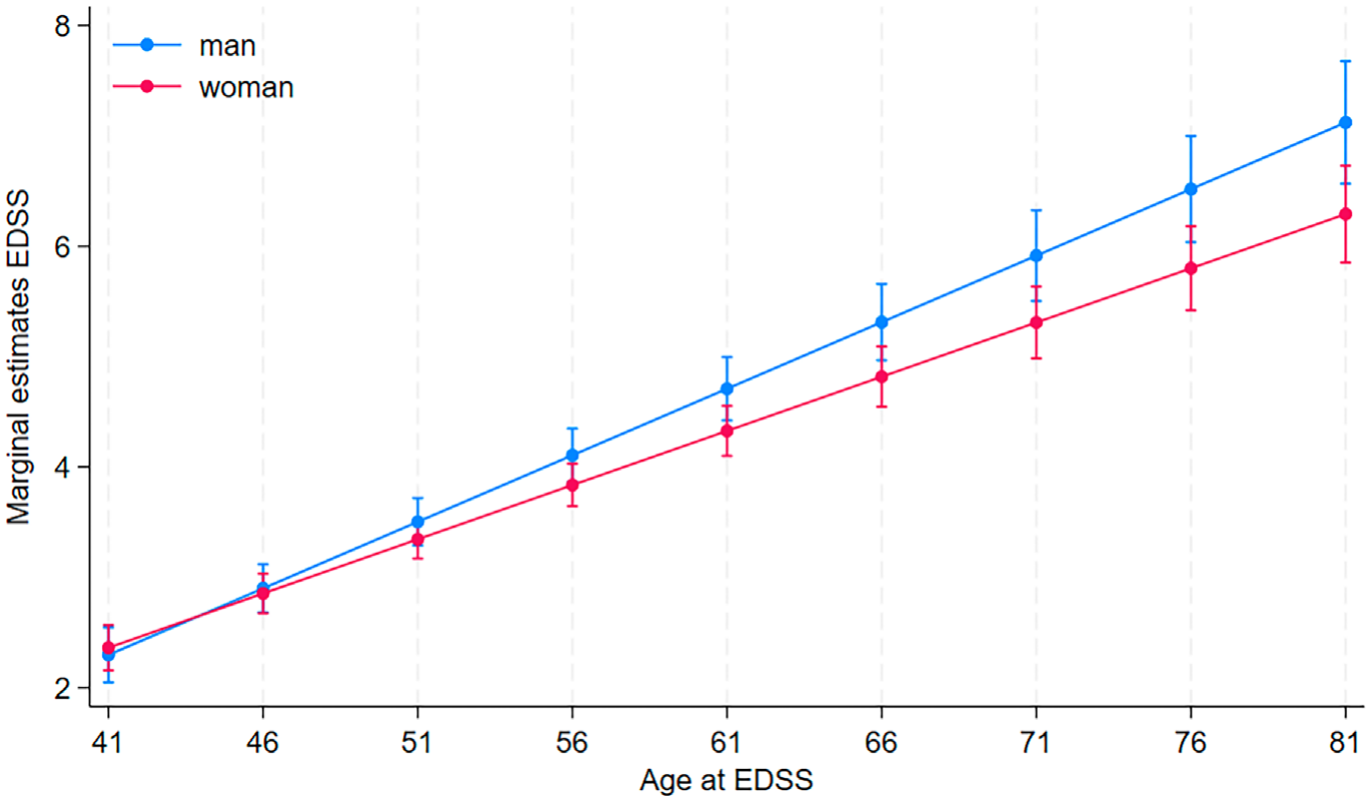
EDSS progression in women and men by age.

**FIGURE 5:**
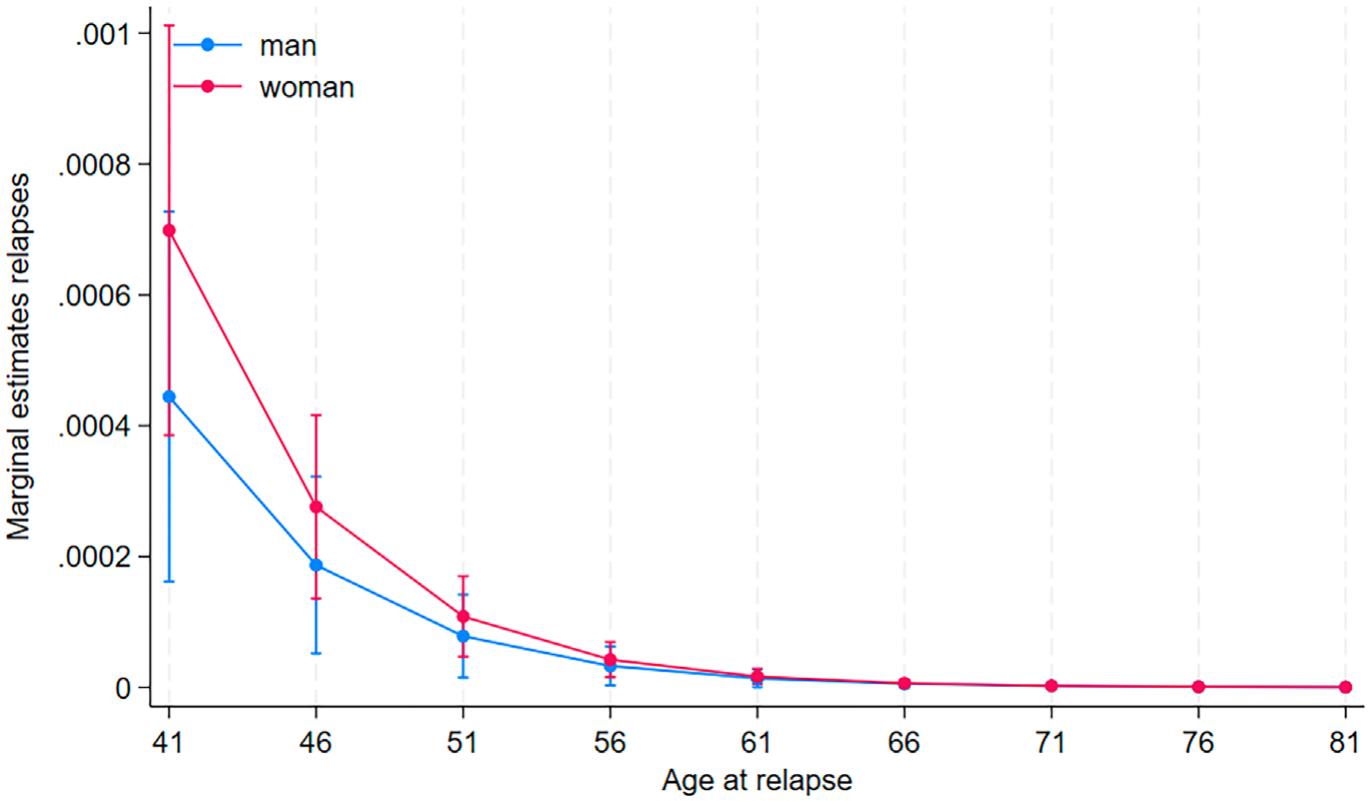
Annual relapse rate in women and men by age.

EDSS increases significantly with age, but the progression differs between men and women, with a slower EDSS progression in women (coef. −0.02, 95% CI −0.04 to −0.002, *p* = 0.032). This remains unchanged even after adjusting for chronological age, disease duration, progressive disease at onset, smoking, treatment with high efficacy DMT treatment and parity, see Figure 4. There was no significant difference in ARR (coef. −0.02, 95% CI −0.04 to 0.004, *p* = 0.107), neither before nor after adjustment, see Figure 5.

### Late‐onset multiple sclerosis (LOMS)

Compared to women with adult‐onset MS, LOMS women were less likely to have optic neuritis at onset (11% vs. 22%, *p* = 0.033), more likely to have motor symptoms (30% vs. 18%, *p* = 0.021) and more likely to have progressive disease at onset (23% vs. 8%, *p* < 0.001). Time from onset to diagnosis was 2.4 years (2.8) in LOMS women versus 7.4 years (8.0) in women with adult‐onset MS.

Compared to LOMS men, LOMS women had significantly more sensory symptoms at onset (37% vs. 13%, *p* = 0.004), less progressive disease at onset (23% vs. 48%, *p* = 0.005) and lower EDSS in 2018 (3.4 (SD 1.8) vs. 4.2 (2.0), *p* = 0.026) and MSSS in 2018 (4.4 (SD 2.5) vs. 5.4 (SD 2.5), *p* = 0.009). Of note, LOMS women went 2.4 years from onset to diagnosis, while men went 4.1 years, though this was not statistically significant (*p* = 0.057). See Table S3.

We found similar findings when comparing only men with LOMS and women with menopause before MS onset (data not shown). There was no significant difference in years from onset to diagnosis (3.1 (SD 3.1) and 4.1 (SD 8.0), *p* = 0.167) in this group.

## DISCUSSION

In this paper, we show that there is no significant difference in EDSS progression before compared to after menopause. In fact, when comparing EDSS progression in men and women, men have a steeper progression curve than women after menopause. This suggests that there is no worsening of EDSS progression following the drop in oestrogen seen in menopause.

Similarly, Otero‐Romero et al. did not find a significant inflection point in the EDSS trajectory around menopause. Nor did early menopause impact the EDSS trajectory [19]. Ladeira et al. found no change in EDSS worsening in a cohort of 37 postmenopausal women [18]. However, other studies have shown disability worsening following menopause. In an Italian cohort with 148 postmenopausal women with MS, the EDSS increased significantly more after menopause. Another study on a cohort following 124 women with MS through menopausal transition, found an increase in EDSS progression from 0.051 units per year before menopause to 0.13 units per year after menopause [17]. What separates our population from these, apart from size, is not the EDSS progression after menopause, which is very similar, but rather a slightly faster EDSS progression before menopause in our population.

The EDSS scale may not be sensitive enough to detect the subtle neuropathological changes associated with menopause, especially not after EDSS 4, when it primarily measures mobility [24]. A survey from 2015 showed that postmenopausal status, surgical menopause and earlier age at menopause were all significantly associated with worse patient reported severity scale (MSRS) scores, even after adjusting for age, disease type and duration [26].

### Relapses after menopause

There was no significant reduction in ARR after menopause in our study. Nor was there a significant difference in relapses by age in the men compared to women. Other studies have found a significant reduction in ARR following menopause [18]. Lorefice et al. found a lower rate of ARR and MRI activity in menopausal women compared to non‐menopausal women [27]. Baroncini et al. reported a significant decrease in ARR, though they also found that younger pwMS with a short‐lasting disease did not have a significant decrease in ARR after menopause, suggesting that the main drivers of ARR reduction in this population were ageing and disease duration rather than menopause itself [16]. A Danish registry study did not find a sex difference in the ARR after the age of 50 [5]. In a metanalysis, there was no significant difference between ARR before and after menopause [20]. Females with systemic lupus erythematous (SLE) have less exacerbations, but a greater accumulation of damage in the affected organs from exacerbations in the postmenopausal period. Menopause may cause an increase in psoriasis exacerbations and a worsening of rheumatoid arthritis progression [7].

Contemporary pwMS differ in many ways from older pwMS, both in that modern pwMS have access to more efficacious DMTs and more rehabilitation, they have overall better health and lifestyle. This may impact the clinical course in contemporary pwMS and, in part, explain the differences seen in our study compared to other studies on disease progression in menopause [28]. Our population was included in 2018, before the introduction of high efficacy therapy as a first therapy, and they were on average 61 years of age at that time. Only 18% had been treated with high efficacy DMTs throughout their disease duration. Including months on high efficacy therapy as a variable did not alter our findings.

### Shorter time for onset to diagnosis if menopause before onset

Women who reached menopause *before MS symptom onset* were diagnosed 4.3 years earlier than women who reached menopause *after MS symptom* onset. In addition, pwMS who reached menopause before onset were more likely to have pyramidal symptoms and progressive disease at onset, similar to the male controls. The EDSS at diagnosis and current MSSS were also significantly higher in pwMS with menopause before onset.

Menopause itself may alter immune, inflammatory and neurodegenerative aspects of MS, contributing to, and exacerbating, the disease [28]. Menopause is associated with cognitive decline and more grey matter atrophy, often referred to clinically as ‘brain fog’ [10]. Testosterone levels in men, which also have a neuroprotective effect [29], start decreasing from the age of 30 to 40 years, named andropause. However, unlike the precipitous drop in hormones that women experience at menopause, the decline in men is gradual, averaging just over 1% a year [30]. Low testosterone levels are associated with worse clinical outcomes [31] and may account for the differing phenotypes in the two sexes [32]. Men with MS have more progressive disease, more brain atrophy, more cognitive dysfunction and more pyramidal symptoms than women with MS [6]. In our population, the men had more progressive disease and more pyramidal symptoms at onset. In addition, although females with adult‐onset MS have a longer time to EDSS 6 compared to males, there is no difference in LOMS [3]. One study reported that Danish men accrue more disability than women after the age of 45 years, though EDSS progression in late onset MS is similar between men and women [5]. Premature menopause is associated with earlier onset of progressive multiple sclerosis in women [33]. In effect, the sudden drop in oestrogen in menopausal pwMS may cause a shift towards a phenotype more similar to that of contemporary male pwMS [34], aligning the EDSS progression in both sexes, thus potentially leading to MS symptoms reaching the symptomatic threshold sooner, allowing for a faster diagnosis.

An alternative explanation is that age, not menopause per se, is the reason for the short time from onset to diagnosis. When comparing women and men with LOMS, there was no significant difference in time from onset to diagnosis. LOMS women had more objective findings at onset (optic neuritis and motor symptoms) compared to women with adult‐onset MS, suggesting they may be easier to diagnose faster. Pagnotti et al. found a diagnostic latency in people with LOMS versus adult‐onset MS [2.6 ± 3.9 vs. 6.5 ± 8.5; *p* < 0.001]. They suggested the reason for this was that people with LOMS are more vulnerable to cognitive and physical decline, thus accelerating the diagnostic process. However, they did not stratify by sex [35].

### Delay in diagnosis

The mean time from onset to diagnosis was 3.3 years longer in pwMS who reached menopause before being diagnosed with MS compared to pwMS who reached menopause. One explanation for the diagnostic delay in postmenopausal women could be the overlap of menopausal symptoms and MS symptoms [21, 26, 36]. In perimenopausal women, both physician and pwMS may believe that all new symptoms are due to menopause. There is a common misconception that MS is a disease of the young, though we have previously shown that a quarter of all pwMS diagnosed after 2006 were 50 years or older [23]. Interestingly, 30% of women started, stopped or changed DMTs the year of menopause. Women and clinicians may attribute symptoms of menopause to side effects of DMTs or signs that the MS is active.

### Strength and weaknesses

The main strength of this study is the population size. Most studies on MS and menopause have smaller cohorts than the current study (*n* = 37–148) [16, 17, 18, 19]. In addition, we have used a real‐world, population‐based cohort with pwMS living in a geographically well‐defined area, but with large variations in socio‐economic factors. This improves the external validity of our results. In addition, we have included a male control group.

The age of menopause in women with MS seem to align with that of the general population, though it is still not clear whether ovarian function is influenced by MS status [14]. We found an age of menopause of 48.3 years, which is lower than the Norwegian population at 52 years [37]. This may be due to information bias as our questionnaire did not ascertain whether menopause was natural or surgical. Our finding is similar to that of Otero‐Romero et al. who found an age of menopause, including surgical menopause, of 47.2 years [19], and Lorefice et al., who found a mean age of 48.5 years [27]. As is the case with most studies on menopause, there will be some recall bias as we rely on the pwMS's retrospective documentation of menopause. However, women's own perceptions of the menopause are based on symptoms, not the arbitrary definition of no menstruation for 12 months, which may also be impacted by birth control. The hormones start to fluctuate and decrease years before this, and consequentially, if there is an impact on MS pathogenesis, it is likely to occur already in the perimenopause [34]. Self‐rated menopausal status appears to relate more closely to a women's endocrine status than definitions based purely on menstrual cycle characteristics [38]. Very few of the pwMS in this study had been on mitoxantrone and none had been through haematopoietic stem cell therapy, both of which may impact ovulation and timing of menopause [39, 40]. We did not have information on other comorbidities, which may impact EDSS progression and relapses in an ageing population [41].

Another limitation is that we did not collect information on HRT, which may impact the effect of menopause. According to a Danish registry study, hormone replacement therapy did not affect disability accrual in menopausal women with MS [42]. We had information on parity, which affects timing of menopause. In addition, we did not include MRI findings, which would have added significant information on disease activity. However, this is the case for most retrospective observational studies.

## CONCLUSION

In conclusion, there were no significant differences in EDSS progression or annual relapse rate in the 5 years before compared to the 5 years after menopause. In fact, age‐matched men progressed faster than women after menopause. Women who reach menopause before their first MS symptom are diagnosed faster. Whether this is due to a sudden drop in hormones or just advanced age remains unclear. Conversely, women who reach menopause before an MS diagnosis experience a diagnostic delay, possibly due to an overlap of symptoms.

## AUTHOR CONTRIBUTIONS

**Cecilia Smith Simonsen:** Conceptualization; investigation; funding acquisition; writing – original draft; methodology; validation; visualization; writing – review and editing; formal analysis; project administration. **Heidi Øyen Flemmen:** Conceptualization; investigation; writing – review and editing. **Line Broch:** Conceptualization; investigation; writing – review and editing. **Harald Myklebust:** Conceptualization; investigation; writing – review and editing. **Pål Berg‐Hansen:** Conceptualization; investigation; writing – review and editing. **Cathrine Brunborg:** Conceptualization; investigation; writing – review and editing; formal analysis. **Elisabeth Gulowsen Celius:** Conceptualization; investigation; writing – review and editing; resources.

## CONFLICT OF INTEREST STATEMENT

This paper was funded by a grant from Vestre Viken Health trust. CSS has received unrestricted research grant from Sanofi and Novartis, advisory board and/or speaker honoraria from Sanofi, Merck, Novartis, BMS and Biogen Idec. HØF has received speaker honoraria from Biogen. LB has received an unrestricted grant from Sanofi Genzyme and advisory board honoraria and/or speaker honoraria from Sanofi Genzyme and Merck. HM has no conflicts of interest to report. PBH has received funding for travel or speaker's fees from Novartis, UCB, Biogen, Teva and Sanofi. CB has no conflicts of interest to report. EGC has received personal compensation for serving on scientific advisory boards and/or speaker honoraria from Almirall, Biogen, BMS, Janssen, Merck, Novartis, Roche, Sanofi and Teva. Her department has received unrestricted research grants from Biogen, Novartis and Genzyme.

## Supporting information


Table S1.

Table S2.

Table S3.

## Data Availability

The data that support the findings of this study are available on request from the corresponding author. The data are not publicly available due to privacy or ethical restrictions.
