# Supplementary material for: The influence of menopause on multiple sclerosis
Source: Eur J Neurol. 2024 Nov 27;32(1):e16566. doi: 10.1111/ene.16566 (PMC11625929; doi:10.1111/ene.16566)
Supplement: Supplementary file 1 — Table S1. [file ENE-32-e16566-s001.pdf]

Supplementary table 1

**All patients, n=**	**Women 559**	**Men 386**	***p***
Ever treated, %	46	51	*0.106*
Number of DMTs, mean (SD)	1.9 (1.1)	1.9 (0.9)	*0.611*
Months on DMTs, mean (SD)	37.4 (56.1)	39 (55.7)	*0.670*
Ever treated with moderate efficacy DMTs, %	41	45	*0.233*
Number of moderate efficacy DMTs, mean (SD)	1.4 (0.9)	1.3 (0.8)	*0.149*
Months on moderate efficacy DMT, mean (SD)	26.6 (45.3)	25.6 (44.4)	*0.745*
Ever treated with high efficacy DMTs, %	18	24	*0.037*
Number of high efficacy DMTs	0.5 (0.8)	0.6 (0.7)	*0.363*
Months on high efficacy DMT, mean (SD)	10.8 (29.1)	13.4 (31.0)	*0.199*
Treated with HSCT, %	2	2	*0.638*
Age HSCT, mean (SD)	50.7(4.5)	54.8 (7.8)	*0.255*
Years from menopause, mean (SD)	6.1(6.2)	-	
Treated with mitoxantrone, %	3	2	*0.243*

**Menopausal women**	
Women on treatment at time of menopause, n= 115, %	21
Moderate efficacy DMT, n=72, %	62
High efficacy DMT, n=43, %	37
Changed/started/stopped DMT in year of menopause, n=35, %	30
Mean number moderate efficacy DMT after menopause, mean (SD)	0.8 (0.7)
Mean number high efficacy DMT after menopause, mean (SD)	0.7 (0.8)
Women not on treatment at time of menopause, n=444, %	79
Started new DMT, n=112, %	25
Months from menopause to start DMT, mean (SD)	5.5 (4.8)
Mean number moderate efficacy DMT after menopause, mean (SD)	0.3 (0.3)
Mean number high efficacy DMT after menopause, mean (SD)	0.1 (0.1)

Supplementary table 1*: Disease modifying therapies (DMT) in men vs women and in the peri-/postmenopausal period. HSCT haematopoietic stem cell transplantation, SD standard deviation*
