# Supplementary material for: The influence of menopause on multiple sclerosis
Source: Eur J Neurol. 2024 Nov 27;32(1):e16566. doi: 10.1111/ene.16566 (PMC11625929; doi:10.1111/ene.16566)
Supplement: Supplementary file 2 — Table S2. [file ENE-32-e16566-s003.pdf]

Supplementary table 2

	Yes	No	*p*
Ever-smoker (SD)	48.2 (4.5)	48.6 (4.8)	*0.197*
Ever treated with any DMT (SD)	47.7 (4.7)	48.8 (4.4)	*0.002*
• Of pwMS treated with DMT, ever-treated with high efficacy DMT (SD)	47.4 (4.5)	47.9 (4.8)	*0.232*
Ever-treated with mitoxantrone (SD)	47.6 (5.6)	48.3 (4.5)	*0.252*
• Of pwMS treated with DMT, ever-treated with mitoxantrone (SD)	47.6 (5.6)	47.7 (4.6)	*0.458*
Treated at the time of menopause	48.1 (4.4)	48.4 (4.6)	*0.299*
Given birth (SD)	48.6 (4.5)	47.3 (4.7)	*0.005*

Supplementary table 2: *Mean age of menopause in ever-smokers (yes) vs never-smokers (no), in ever-treated with DMT (yes) vs never-treated with DMT, treated at the time of menopause vs not treated at the time of menopause and pwMS who had ever given birth (yes) vs never given birth (no). SD standard deviation, DMT disease modifying therapy*
