# Supplementary material for: The influence of menopause on multiple sclerosis
Source: Eur J Neurol. 2024 Nov 27;32(1):e16566. doi: 10.1111/ene.16566 (PMC11625929; doi:10.1111/ene.16566)
Supplement: Supplementary file 3 — Table S3. [file ENE-32-e16566-s002.pdf]

Supplementary table 3

	LOMS women	LOMS men	*p*
N (%)	74	47	
Age, years mean(SD)	64.8 (7.3)	65.7 (6.1)	*0.248*
EDSS at diagnosis mean(SD)	3.0 (1.5)	2.9 (1.1)	*0.482*
Age at onset, mean (SD)	53.8 (4.1)	53.4 (7.1)	*0.326*
Age at diagnosis, mean (SD)	56.3 (5.3)	57.4 (5.5)	*0.125*
Time from onset to diagnosis, mean (SD)	2.4 (2.8)	4.1 (8.0)	*0.057*
Sensory symptoms at onset, %	37	13	*0.004*
Optic neuritis at onset, %	11	9	*0.690*
Pyramidal symptoms at onset, %	30	44	*0.138*
Progressive disease at onset, %	23	47	*0.020*
Multiple symptoms at onset, %	40	49	*0.394*
Present or past smokers, %	68	78	*0.206*
Live births, mean (range)	1.9	1.9	*0.389*
Ever-treated DMT, %	37	30	*0.448*
Ever-treated high efficacy DMT, %	14	13	*0.906*
Ever treated with mitoxantrone, %	0	0	
Treatment at time of menopause, %	1 woman		
Age at menopause, mean (SD)	48.6 (4.2)		
Age at menarche, mean (SD)	13.1 (1.4)		
Years from menarche to menopause, mean (SD)	35.5 (4.5)		
Years from onset to menopause, mean (SD)	5.2 (6.1)		
EDSS 2018, mean (SD)	3.4 (1.8)	4.2 (2.0)	*0.026*
MSSS 2018, mean (SD)	4.4 (2.5)	5.4 (2.5)	*0.009*

Supplementary table 3: *Demographics and clinical data in female and male PwMS with LOMS. EDSS expanded disability status scale, SD standard deviation, DMT disease modifying therapy MSSS multiple sclerosis severity score*
